# Teaching triage in disaster medicine – same subject, but different approach

**DOI:** 10.1186/s13049-025-01322-5

**Published:** 2025-01-16

**Authors:** Amir Khorram-Manesh

**Affiliations:** 1 Department of Surgery, Institute for Clinical Sciences, Sahlgrenska Academy, Gothenburg University, Gothenburg, Sweden; 2 Center for Disaster Medicine, Sahlgrenska Academy, Gothenburg University, Gothenburg, Sweden; 3 Gothenburg Emergency Medicine Research Group (GEMREG), Sahlgrenska University Hospital, Gothenburg, Sweden

**Keywords:** Disaster, Education, Emergency, Mass casualty, Multicasualty, Triage

## Abstract

**Background:**

Disaster management is an inter-, intra-, and cross-disciplinary task in which different specialties partake. Triage is a crucial part of disaster education. A synchronized approach and mutual understanding of triaging and agreement on priorities are essential for saving lives.

**Case study:**

Educational initiatives in disaster medicine aim to address issues that highlight the differences between more routine multi-casualty incidents and rarer mass casualty incidents. These differences are characterized by the number of victims, available resources, and environmental factors that may jeopardize the safety of victims and healthcare providers. While routine triage algorithms are often used in multiple casualty emergencies, considering environmental factors in mass casualty incidents caused by natural or human-made hazards should be equally important.

**Conclusions:**

The impacts of environmental factors are usually not discussed in disaster medicine education, resulting in professionals having difficulties understanding the limitations of implementing routine triage algorithms during disaster response.

## Backgrounds

Triage is an important part of emergency management that begins at the incident scene, with primary triage determining treatment and transport priorities, followed by secondary triage at the casualty clearing station and before hospital transport [1]. Mass casualty incidents (MCIs) are rare incidents that differ from more routine multi-casualty incidents in terms of two main factors: (1) the number of casualties and available resources, requiring MCI triage to differentiate between noncritically and critically injured casualties in a situation with demanding time and resource accessibility, and (2) the environment in which these events occur, with the MCI occurring in a potentially threatening environment either due to natural or man-made hazards [2, 3].

The unpredictable nature of the MCI calls for an “all-hazards” approach not only for the sake of patients but also for the safety of healthcare providers. One way to improve triage skills and overcome situation-dependent challenges is to practice diverse algorithms under different circumstances to learn about various hazards and their impacts on victims and healthcare providers.

## Mass casualty triage vs. multi-casualty triage

Disaster medicine is a discipline that encompasses emergency medicine and disaster management as well as the unique medical requirements of a community during a disaster. Triage is an essential part of disaster medicine education and prioritizes victims in an environment characterized by resource scarcity and associated with ethical challenges and complexities. Although triage is an important subject, it is theoretically learned via different triage algorithms, which are designed mainly for peacetime, routine multi-casualty, and small incidents [4, 5]. While military or disaster triage is performed in riskier and threatful situations due to natural or man-made hazards that can compromise victims and healthcare providers, routine triage can be performed in often safe areas with less risk for life-threatening situations [6]. Consequently, even though they are skilled and professional in triaging, learners do not recognize other factors affecting their decision-making in a more threatening situation [5].

Triaging in a disaster environment is affected by threats that continuously influence the dynamic nature of triaged patients and the safety of healthcare providers. This means that some of the steps in a triage algorithm may not be possible, and an alternative approach should be advocated [7, 8]. One such step is labeling patients in black tags, representing either dead or, in some countries, lifeless patients. In many countries, a physician should assess a victim and declare the death, making it difficult to do so during primary triage in a major incident. To overcome this challenge, some algorithms label these patients as lifeless. Lifeless patients are usually re-evaluated before the primary triage period is over [2].

In these situations, the initial approach is to perform life-saving maneuvers, selecting victims with a greater chance of living for detailed triage and assessment in the next step. In a routine multi-casualty incident with a safe environment, there is enough time to make a proper decision according to the algorithm used, label the victims, and plan to reevaluate them later [1–3]. In contrast, limited time and an unsafe and threatening environment do not allow a reevaluation of lifeless patients or a declaration of deceased victims. Additionally, many learn to follow the algorithm without considering reverse triage in necessary situations.

Differentiating triage in multi-casualty and mass casualty situations from a time and hazard perspective is crucial in teaching disaster triage since, under threatening conditions, victims may preferably be evacuated first, regarded as patients with Red priority, in need of immediate measures, to be prioritized later in a safer area with more time to assess their medical condition and perform life-saving interventions [8]. This is fully understandable since the time for a primary assessment of all victims is not enough; there are many hazards, and each can jeopardize the lives of victims and healthcare providers. In addition, the evacuation of all victims offers the possibility to reevaluate the victims’ medical condition [9, 10].

Conflicts, natural hazard-induced disasters, fires, and situations in which the risk for explosion or terror attacks remains are situations in which time and safety should be prioritized before the triage algorithm is followed blindly [9, 10].

## Conclusions

Teaching triage, especially primary triage in disaster and mass casualty situations, should focus not only on algorithms but also on obtaining wider knowledge of situational awareness. Situational awareness and risk assessment are crucial in determining the pace and order of triage in mass casualty situations. Evacuating patients without primary triage might be a valid and necessary approach in threatening situations.

## Data Availability

No datasets were generated or analysed during the current study.

## Abbreviations

MCI: Mass casualty incident
